# Deficiency of the Purinergic Receptor 2X_7_ Attenuates Nonalcoholic Steatohepatitis Induced by High-Fat Diet: Possible Role of the NLRP3 Inflammasome

**DOI:** 10.1155/2017/8962458

**Published:** 2017-11-15

**Authors:** Claudia Blasetti Fantauzzi, Stefano Menini, Carla Iacobini, Chiara Rossi, Eleonora Santini, Anna Solini, Giuseppe Pugliese

**Affiliations:** ^1^Department of Clinical and Molecular Medicine, “La Sapienza” University, Via di Grottarossa 1035, 00189 Rome, Italy; ^2^Department of Clinical and Experimental Medicine, University of Pisa, Via Roma 67, 56126 Pisa, Italy; ^3^Department of Surgical, Medical, Molecular and Critical Area Pathology, University of Pisa, Via Roma 67, 56126 Pisa, Italy

## Abstract

Molecular mechanisms driving transition from simple steatosis to nonalcoholic steatohepatitis (NASH), a critical step in the progression of nonalcoholic fatty liver disease (NAFLD) to cirrhosis, are poorly defined. This study aimed at investigating the role of the purinergic receptor 2X_7_ (PR2X_7_), through the NLRP3 inflammasome, in the development of NASH. To this end, mice knockout for the *Pr2x_7_* gene (*Pr2x_7_*^−/−^) and coeval wild-type (WT) mice were fed a high-fat diet (HFD) or normal-fat diet for 16 weeks. NAFLD grade and stage were lower in *Pr2x_7_*^−/−^ than WT mice, and only 1/7 *Pr2x_7_*^−/−^ animals showed evidence of NASH, as compared with 4/7 WT mice. Molecular markers of inflammation, oxidative stress, and fibrosis were markedly increased in WT-HFD mice, whereas no or significantly reduced increments were detected in *Pr2x_7_*^−/−^ animals, which showed also decreased modulation of genes of lipid metabolism. Deletion of *Pr2x_7_* gene was associated with blunted or abolished activation of NLRP3 inflammasome and expression of its components, which were induced in liver sinusoidal endothelial cells challenged with appropriate stimuli. These data show that *Pr2x_7_* gene deletion protects mice from HFD-induced NASH, possibly through blunted activation of NLRP3 inflammasome, suggesting that PR2X_7_ and NLRP3 may represent novel therapeutic targets.

## 1. Introduction

Nonalcoholic fatty liver disease (NAFLD) has become the leading cause of chronic liver disease worldwide [1], as a result of the epidemics of obesity and type 2 diabetes. It encompasses a wide spectrum of disease conditions, from simple steatosis to nonalcoholic steatohepatitis (NASH), cirrhosis, and hepatocellular carcinoma [2]. Transition from steatosis to NASH is characterized by superimposition of inflammation and hepatocyte degeneration and death, ultimately leading to tissue fibrosis [3]. Lipotoxicity from nontriglyceride fatty acid metabolites is now recognized as a central mechanism driving hepatic inflammation and injury. When flux of free fatty acids (FFAs) from diet, adipose tissue lipolysis, and hepatic de novo lipogenesis is higher than the rate of FFA oxidation or incorporation into triglycerides for storage as lipid droplets or export as VLDL, exceeding FFAs can generate lipotoxic intermediates which may induce oxidative stress, endoplasmic reticulum stress, mitochondrial dysfunction, and overproduction of proinflammatory cytokines and adipokines [4, 5]. However, the precise molecular mechanism triggering steatosis progression to NASH has not been elucidated yet.

A growing body of evidence indicates a major role for the purinergic system, particularly extracellular ATP (eATP) signaling through the purinergic receptor 2X_7_ (PR2X_7_), in several inflammatory and fibrotic disorders including NASH [6, 7]. In detail, Das et al. showed that *Pr2x_7_* gene deletion was associated with protection from liver injury in two rodent models of NASH: the toxin-induced model, using coadministration of a high-fat diet (HFD) and a low-dose environmental toxin bromodichloromethane, and the diet-induced model, using a methionine choline-deficient (MCD) diet [8]. Likewise, Hoque et al. reported that mice knockout for *Pr2x_7_* gene (*Pr2x_7_*^−/−^) as well as wild-type (WT) mice treated with the specific PR2X_7_ antagonist A438079 exhibited significantly decreased acetaminophen hepatotoxicity [9]. Antagonism of PR2X_7_ with A438079 and Brilliant blue G was also found to attenuate liver fibrosis induced by carbon tetrachloride in mice [10] and common bile duct ligation in rats [11], respectively.

Stimulation of PR2X_7_ by eATP results in rapid opening of a ligand-gated cation channel, followed by induction of a cytoplasmic pore via pannexin-1, which triggers K^+^ efflux and allows danger signals to access the cytosol and activate the nucleotide-binding and oligomerization domain (NOD), leucine-rich repeat, and pyrin domain containing (NLRP) 3 inflammasome [12]. The NLRP3 inflammasome is expressed primarily in macrophages and dendritic cells [13], but also in nonhematopoietic tissues, including the liver at the level of hepatocytes, Kupffer cells, endothelial cells, stellate cells, and myofibroblasts [14]. This multiprotein platform is composed of an inflammasome sensor molecule, containing an N-terminal pyrin domain (PYD) and a caspase recruitment domain (CARD). Once activated, it oligomerizes and recruits the PYD-CARD adaptor protein apoptosis-associated speck-like protein containing a CARD (PYCARD or ASC) and the protease procaspase-1. Caspase-1 auto-activation results in the cleavage of prointerleukin- (IL-) 1*β* and IL-18 into their mature form, which are released [13]. In addition to mediating inflammation via canonical IL-1*β* and IL-18-dependent mechanisms, the NLRP3 inflammasome regulates cell death through noncanonical caspase-1-dependent and independent pathways leading to pyroptosis and pyronecrosis, respectively [15].

We have previously shown that deletion of *Pr2x_7_* gene attenuated renal disease in mice fed a HFD and that this was associated with blunted upregulation of the NLRP3 inflammasome components NLRP3, ASC, procaspase-1, pro-IL-1*β*, and pro-IL-18 and reduced the formation of mature caspase-1 [16]. The observation that PR2X_7_ was required for eATP-stimulated IL-1*β* release in Kupffer cells supports the hypothesis that also in the liver the effects of PR2X_7_ stimulation are mainly mediated by NLRP3 activation [9]. Indeed, numerous experimental observations suggest an involvement of NLRP3 in transition from steatosis to NASH [14]. Treatment with the NLRP3 inflammasome blocker MCC950 attenuated liver inflammation and fibrosis in two mouse models of NASH, that is, the *Alms1* mutant (*foz/foz*) mice fed with an atherogenic diet and C57BL/6 mice fed with an MCD diet [17]. Likewise, deletion of the *NLRP3* gene protected from NASH induced by choline-deficient amino acid-defined diet [18], though another report found exacerbated MCD diet-induced hepatic steatosis and inflammation mediated by gut microbial dysbiosis [19]. Conversely, *Nlrp3* knockin mice showed accelerated NASH [18, 20], associated with marked hepatocyte pyroptosis [20].

This study aimed at investigating the effect of *Pr2x_7_* gene deletion on the development of experimental NASH induced by HFD in mice.

## 2. Methods

### 2.1. Design

The study design consisted of (a) *in vivo* studies, in which we assessed whether disruption of the *Pr2x_7_* gene attenuates NASH via blunted activation of the NLRP3 inflammasome, and (b) *in vitro* studies, in which we assessed whether PR2X_7_-dependent activation of the NLRP3 inflammasome occurs also at the level of resident liver cells. In the *in vivo* studies, adult (aged six weeks), male *Pr2x_7_*^−/−^and coeval WT mice were used. The *Pr2x_7_*^−/−^ mice were of the two currently available strains, that is, the Pfizer strain, backcrossed onto a B6D2 background, which has a neomycin cassette (Neo) inserted into exon 13 [21], and the GlaxoSmithKline strain, backcrossed onto a C57BL6 background, which has a LacZ gene and Neo inserted into exon 1 [22]. The *Pr2x_7_*^−/−^ mice of Pfizer strain were purchased from Jackson Laboratory (Bar Harbor, ME), whereas those of the GlaxoSmithKline strain were kindly provided by Professor Francesco Di Virgilio (University of Ferrara, Ferrara, Italy). The reason for using the two strains is that splice variants with reduced function may escape inactivation in selected tissues from both the Pfizer [23] and the GlaxoSmithKline [24] strain. Mice were housed in a germ-free stabularium in accordance with the Principles of Laboratory Animal Care (NIH Publication number 85–23, revised 1985) and with national laws and received water and food ad libitum. The study protocol was approved by the locally appointed ethics committee. Mice from both genotypes were fed for 16 weeks either a HFD (DIO diet D12492, 60% of total calories from fat) or a normal-fat diet (NFD, DIO diet D12450B; 10% of total calories from fat), purchased from Research Diets (Mucedola, Settimo Milanese, Italy). The four groups consisted of 12–14 animals each, half of the Pfizer strain and half of the GlaxoSmithKline strain. At the end of the 16-week period, mice were anaesthetized with intraperitoneal ketamine (Imalgene®, 60 mg/kg body weight) and xylazine (Rompum®, 7.5 mg/kg body weight) and a longitudinal incision of the abdominal wall was performed, a blood sample was obtained, and the liver was removed and weighed. Then, a portion of liver tissue was immediately fixed by immersion in phosphate buffered 4% formaldehyde solution and processed for light microscopy examination, morphometrical evaluation, and immunohistochemistry. The remaining liver tissue was frozen in liquid nitrogen and used for extraction of proteins and total RNA [25]. In the *in vitro* studies, human liver sinusoidal endothelial cells (LSECs) were plated onto fibronectin-coated dishes and cultured in endothelial cell medium supplemented with 5% FBS, antibiotics, and endothelial cell growth supplement (ScienCell Research Laboratories, Carlsbad, CA), at 37°C in 95% air-5% CO_2_ humidified atmosphere. Cells were then incubated for 21 hours with serum-free medium containing 100 ng/ml recombinant human tumor necrosis factor- (TNF-) *α* (PeproTech, Rocky Hill, NJ) followed by 0.2 mM 2′(3′)-O-(4-benzoylbenzoyl)ATP (BzATP, Sigma-Aldrich, Saint Louis, MO) for 45 min. Cell lysates were then collected [16]. This stimulus was chosen in order to mimic the sterile inflammation occurring in NASH, consistent with a very recent report showing a key role for TNF-*α* as a mediator of liver inflammation and fibrosis induced by constitutive NLRP3 inflammasome activation in myeloid-derived cells [26].

### 2.2. Liver Morphology/Morphometry

Liver morphology was assessed based on the American Association for the Study of Liver Disease Guidelines [27], as previously reported [25]. Briefly, NAFLD was graded and staged in hematoxylin and eosin-stained sections. NAFLD grading was assessed based on the percentage of parenchyma involved by steatosis (grades 0 to 3 as follows: 0, no fat; 1, <33%; 2, 33–66%; and 3, >66%). The steatosis grade and the presence of inflammation, hepatocyte degeneration (acidophil or Councilman's bodies, ballooning, and Mallory's hyaline) or necrosis, and fibrosis were then considered for NAFLD staging (stages 1 to 4, with stages 3 and 4 corresponding to NASH). Subsequently, samples from mice with NAFLD stage 3 and 4 were graded and staged for NASH. NASH grading was accomplished based on the type of fat (macrovesicular, microvesicular, or mixed) and the extent of inflammation (scored 0 to 3 as follows: 0, no; 1, mild; 2, moderate; and 3, severe) and hepatocyte degeneration or necrosis. NASH staging was performed by assessing the extent and distribution of fibrosis in sections stained with Masson's trichrome.

### 2.3. Biochemistry and ELISA

Blood samples obtained from experimental animals were analyzed for fasting levels of glucose, with the aid of an automated colorimetric instrument (Glucocard™ SM, A. Menarini Diagnostics, Florence, Italy); cholesterol, triglycerides, aspartate transaminase (AST), and alanine transaminase (ALT), by standard chemical methods (VITROS5.1 FS Chemistry System, Ortho-Clinical Diagnostics, Rochester, NY); free fatty acids (FFAs), using the NEFA C kit (Wako, Osaka, Japan); and insulin, by enzyme immunoassay (Ultrasensitive Mouse Insulin ELISA kit, Mercodia AB, Uppsala, Sweden). The homeostasis model assessment-insulin resistance (HOMA-IR) index was then calculated from fasting glucose and insulin [16, 25]. Hepatic levels of eATP were assessed colorimetrically in liver tissue homogenates using the ATP assay kit (ab83355, Abcam, Cambridge, UK) as per manufacturer's instructions.

### 2.4. Immunohistochemistry and Western Blot Analysis

Liver content and distribution of the advanced glycation end product (AGE) N*^ε^*-(carboxymethyl)lysine (CML) and the 47 kDa cytosolic subunit of neutrophil NADPH neutrophil cytosol factor 1 (NCF1, also known as p47phox) were assessed by immunohistochemistry, together with protein expression of PR2X_7_ and NLRP3. The primary and secondary antibodies used are reported in Supplemental Table 1 available online at https://doi.org/10.1155/2017/8962458. Staining was analyzed using an image analysis system (Optimas™6.5, Bioscan, Washington DC) at a fixed color threshold in 20 random fields of the liver at a final magnification of 400x, and results were expressed as the mean percentage of field area occupied by the specific stain [16, 25]. Levels of caspase-1 in the liver and LSECs were evaluated by Western blot analysis and normalized to the levels of *β*-actin. The antibodies used are reported in Supplemental Table 1. Bands were detected by an enzymatic chemiluminescence kit (Immobilon Western, Millipore, Billerica, MA) and quantified by scanning densitometry using a GS-670 Imaging Densitometer (Bio-Rad Laboratories, Hercules, CA) [16].

### 2.5. Quantitative Real-Time PCR

Total RNA was extracted from the liver with the RNAeasy mini kit (Qiagen, Milan, Italy). Then, 1 *μ*g of RNA was reverse-transcribed in a 20 *μ*l reaction tube using high capacity cDNA reverse transcription kit (Applied Biosystems, Monza, Italy). Quantitative real-time PCR (qRT-PCR) was performed in triplicate using a StepOne™ Real-Time PCR instrument (Thermo Fisher Scientific, Monza, Italy) following the standard protocol. The following transcripts were quantified by TaqMan gene expression assays (Applied Biosystems) using the assays reported in Supplemental Table 2: (a) the inflammatory mediators monocyte chemoattractant protein-1 (MCP-1, *Ccl2*), TNF-*α* (*Tnfa*), and interferon-*γ* (*Ifng*); (b) the markers of activated Th1 lymphocytes and murine macrophage activation, CX chemokine receptor 3 (*Cxcr3*) and F4/80 (*Adgre1*), respectively; (c) the regulator of ER stress-mediated apoptosis CCAAT/enhancer-binding protein (*C/EBP*) homologous protein (CHOP, *Ddit3*); (d) the receptor for AGEs (RAGE, *Ager*); (e) the extracellular matrix proteins fibronectin (*Fn1*) and collagen I (*Col1a1*) and the profibrotic cytokine transforming growth factor-*β*1 (*Tgfβ1*); (f) the transcription factors regulating lipid metabolism, sterol regulatory element-binding transcription factor 1c (*Srebf1*), peroxisome proliferator-activated receptor *α* and *γ* (*Ppara* and *Pparg*), and liver X receptor (LRX) *α* and *β* (*Nr1h3* and *Nr1h2*); (g) the enzymes of fatty acid synthesis, acetyl-CoA carboxylase (*Acaca*) and FA synthase (*Fasn*), of fatty acid *β*-oxidation, carnitine palmitoyltransferase I (*Cpt1a*) and acyl-CoA oxidase 1 (*Acox1*), and of cholesterol synthesis, hydroxymethylglutaryl-CoA reductase (*Hmgcr*); (h) the protein responsible for triglyceride transfer to apolipoprotein B100, microsomal triglyceride transfer protein (*Mttp*); and (i) the components of the PR2X_7_-NLRP3 axis, *Pr2x_7_*, *Nlrp3*, *Pycard*, *procaspase-1* (*Casp1*), and *Il1β*. The amount of the target gene was normalized to *β*-actin (*ACTB*). Results were analyzed using the SDS 2.1 Applied Biosystems software [28].

### 2.6. Statistical Analysis

Results are expressed as means ± SD or median and interquartile range and percent change versus controls. Statistical significance was evaluated by (a) Pearson's *χ*^2^ test for categorical variables; (b) Student's *t*-test or one-way ANOVA followed by the Bonferroni correction for multiple comparisons for parametric continuous variables; and (c) the Mann–Whitney *U* test or the Kruskal-Wallis test followed by the Wilcoxon's signed ranks test for nonparametric continuous variables. A *p* value < 0.05 was considered significant. All statistical tests were performed on raw data using the SPSS version 13.0 (SPSS Inc., Chicago, IL).

## 3. Results

### 3.1. Metabolic Parameters and Liver Enzymes

Body weights were significantly higher in mice on a HFD, as compared with animals fed with a NFD. Likewise, blood glucose and insulin concentrations, the HOMA-IR index, and triglyceride, cholesterol, and FFA levels were higher in HFD- versus NFD-fed mice. Interestingly, there was no difference between the two genotypes of the Pfizer strain (Table 1), whereas *Pr2x_7_*^−/−^-HFD mice of the GlaxoSmithKline strain showed no increase in body weight versus *Pr2x_7_*^−/−^-mice and significantly lower increments in glucose, insulin, HOMA-IR, triglycerides, cholesterol, and FFAs than the corresponding WT-HFD animals. To rule out the possibility that blunted metabolic abnormalities per se may have resulted in attenuated hepatic injury in the *Pr2x_7_*^−/−^ mice of the GlaxoSmithKline strain, only data from the Pfizer strain are presented herein, despite the fact that, at variance with metabolic parameters, all measures of liver function and structure were virtually identical between the two strains. Levels of AST and ALT increased significantly in HFD-fed mice from both genotypes, as compared with the corresponding NFD-fed animals, though increases were significantly less marked in *Pr2x_7_*^−/−^ than in WT animals (Table 1).

### 3.2. Grading and Staging of NAFLD and NASH

**Figure 1:**
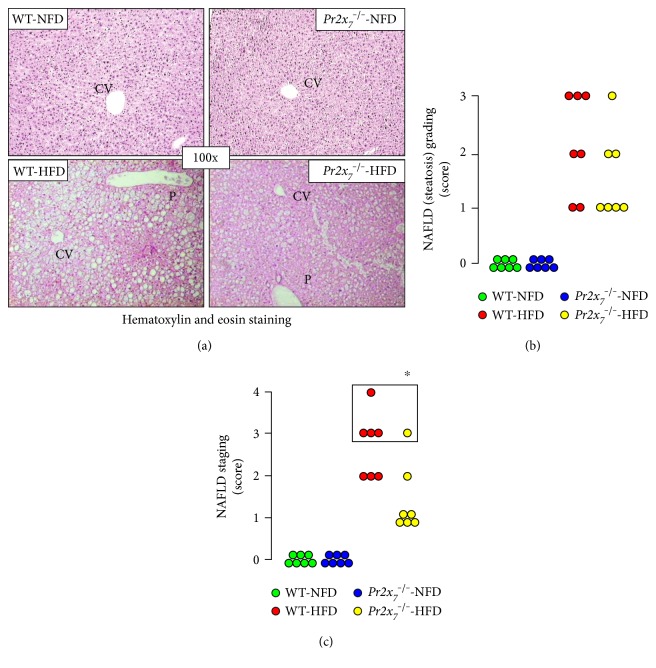
NAFLD grading and staging. Hematoxylin and eosin staining of liver sections from representative animals ((a); original magnification 100x) and NAFLD (steatosis) grading (b) and staging (c) in NFD- and HFD-fed WT and *Pr2x_7_*^−/−^ mice (*n* = 7 per group). P = portal area; CV = centrolobular vein. The box indicates mice fulfilling NASH criteria. ^∗^*P* < 0.05 versus WT mice. NAFLD = nonalcoholic fatty liver disease; NFD = normal-fat diet; HFD = high-fat diet; WT = wild type; *Pr2x_7_*^−/−^ = knockout for purinergic receptor 2X_7_ gene; NASH = nonalcoholic steatohepatitis.

**Figure 2:**
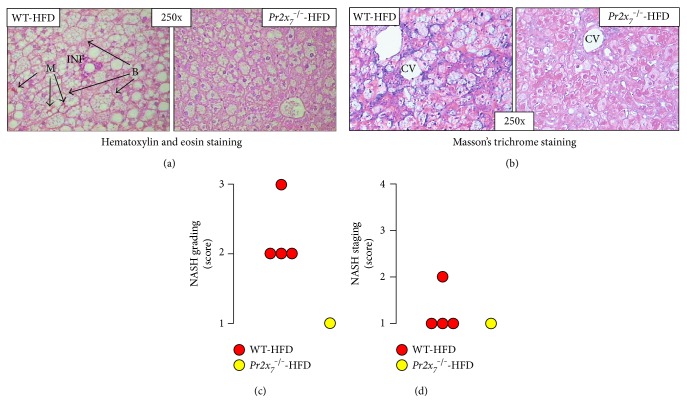
NASH grading and staging. Hematoxylin and eosin ((a); original magnification 250x) and Masson's trichrome ((c); original magnification 250x) staining of liver sections from representative animals; and NASH grading (c) and staging (d) in the HFD-fed WT (*n* = 4) and *Pr2x_7_*^−/−^ (*n* = 1) mice fulfilling NASH criteria. P = portal space; CV = centrolobular vein; B = ballooning degeneration; M = Mallory's body; INF = inflammation. NASH = nonalcoholic steatohepatitis; HFD = high-fat diet; WT = wild type; *Pr2x_7_*^−/−^ = knockout for purinergic receptor 2X_7_ gene.

Steatosis was detected only in HFD-fed mice from both genotypes, though it was of higher grade in WT than in *Pr2x_7_^−/−^* mice (Figure 1(a)). In detail, NAFLD grading showed that three out of 7 WT-HFD mice fell in grade 3 steatosis, whereas four out of 7 *Pr2x_7_^−/−^*-HFD mice were assigned to grade 1 steatosis (Figure 1(b)). Likewise, NAFLD grading showed that, of the WT-HFD mice, three had stage 2 (predominantly microvesicular steatosis with mild lobular inflammation), three had stage 3 (mixed steatosis with lobular inflammation and ballooning degeneration), and one had stage 4 (mixed steatosis with lobular inflammation and ballooning degeneration and macrovesicular steatosis, associated with portal and lobular inflammation, Councilman's bodies, ballooning degeneration, Mallory's hyaline, and fibrosis) NAFLD. Conversely, only one of the *Pr2x_7_*^−/−^-HFD animals fell in stage 3 NAFLD, whereas the remaining six mice did not fulfill NASH criteria, as one had stage 2 and five had stage 1 (simple steatosis) NAFLD (Figure 1(c)). Of the four WT-HFD animals classified as having NASH, three showed grade 2 (moderate) and one grade 3 (severe or florid) NASH, whereas one exhibited stage 2 (zone 3 portal/periportal, perivenular/centrolobular, perisinusoidal/pericellular fibrosis; focal or extensive) and three stage 1 (i.e., as stage 2, except portal fibrosis) fibrosis (Figures 2(a) and 2(b)). The only one *Pr2x_7_*^−/−^ mouse fulfilling NASH criteria was graded 1 (mild) for NASH and staged 1 for fibrosis (Figures 2(c) and 2(d)).

### 3.3. Markers of Liver Inflammation, Fibrosis, and Lipid Metabolism

**Figure 3:**
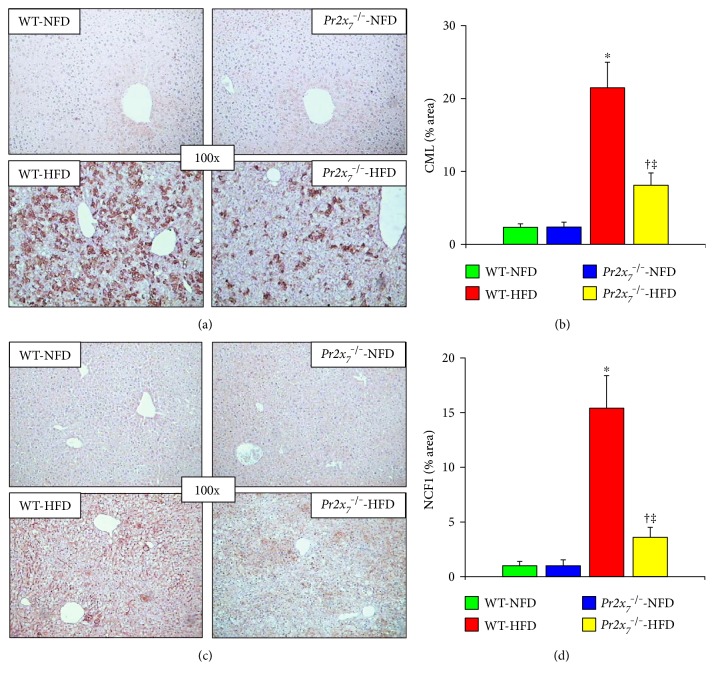
Oxidative and carbonyl stress markers. Immunohistochemistry for CML ((a); original magnification 100x) and NCF1 ((c); original magnification 100x) in liver sections from representative animals and quantification of liver CML (b) and NCF1 (d) protein content in HFD-fed WT and *P2x_7_r*^−/−^ mice (mean ± SD; *n* = 4–6 per group). ^∗^*P* < 0.001 or ^†^*P* < 0.05 versus the corresponding NFD-fed mice; ^‡^*P* < 0.001 versus WT mice. CML = N*^ε^*-(carboxymethyl)lysine; NCF1 = neutrophil cytosol factor 1; NFD = normal-fat diet; HFD = high-fat diet; WT = wild type; *Pr2x_7_*^−/−^ = knockout for purinergic receptor 2X_7_ gene.

The liver content of CML (Figures 3(a) and 3(b)) and NCF1 (Figures 3(c) and 3(d)) was markedly increased in HFD-fed WT and, to a significantly lesser extent, *Pr2x_7_*^−/−^ mice. Transcripts of the markers of inflammation and endoplasmic reticulum and carbonyl stress *Ccl2*, *Tnfa*, *Cxcr3*, *Adgre1*, *Ddit3*, *and Ager*, *but not Ifng*, increased significantly in WT-HFD mice, but not in *Pr2x_7_*^−/−^-HFD animals (Table 2). Likewise, the mRNA expression levels of the extracellular matrix components *Fn1* and *Col1a1* increased significantly in WT, but not *Pr2x_7_*^−/−^ mice on a HFD, whereas those *Tgfβ* increased to a much lesser extent in *Pr2x_7_*^−/−^ than in WT animals (Table 2). In response to the HFD, marked changes were observed in the gene expression level of several transcription factors and enzyme of lipid metabolism. In particular, transcript expression of *Srebf1* and, to a lesser extent, of *Ppara*, *Pparg*, and *Nr1h3* increased in WT-HFD mice, but not or significantly less markedly in *Pr2x_7_*^−/−^-HFD animals. In response to the HFD, also transcripts of *Acaca*, *Fasn*, and *Cpt1* increased only or significantly more in WT, as compared with *Pr2x_7_*^−/−^mice. In contrast, no significant effect of HFD or *Pr2x_7_*^−/−^ gene deletion was observed in the mRNA levels of *Nr1h2*, *Acox1*, *Hmgcr*, and *Mttp* (Table 2).

### 3.4. Liver Expression and Activation of the PR2X_7_/NLRP3 Axis

**Figure 4:**
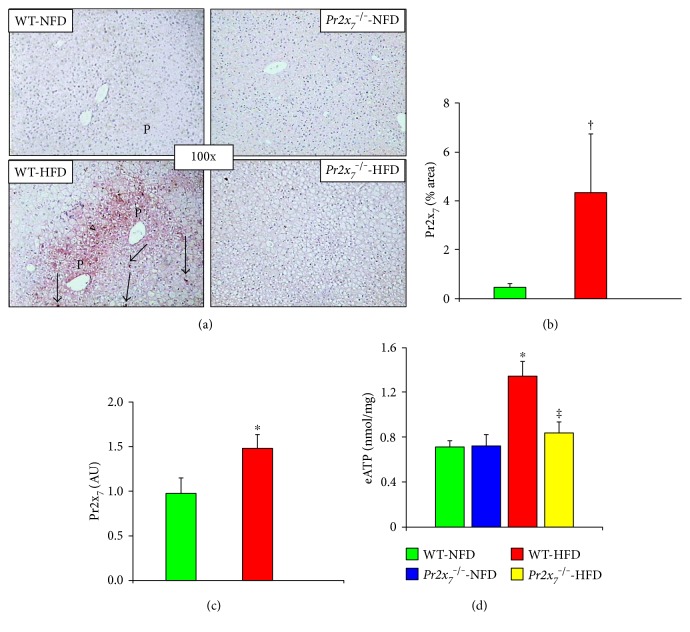
Hepatic PR2X_7_ expression. Immunohistochemistry for PR2X_7_ in liver sections from representative animals ((a); original magnification 100x) and quantification of liver PR2X_7_ protein content (b), *Pr2x_7_* mRNA expression (c), and eATP levels (d) in NFD- and HFD-fed WT and *P2x_7_r*^−/−^ mice (mean ± SD; n = 4–6 per group). P = portal space; > = parenchymal cell (hepatocyte) staining; → = nonparenchymal (sinusoidal lining) cell staining. ^∗^*P* < 0.001 or ^†^*P* < 0.01 versus NFD-fed mice; ^‡^*P* < 0.001 versus WT mice. PR2X_7_ = purinergic receptor 2X_7_; *Pr2x_7_* = PR2X_7_ gene; eATP = extracellular ATP; NFD = normal-fat diet; HFD = high-fat diet; WT = wild type.

**Figure 5:**
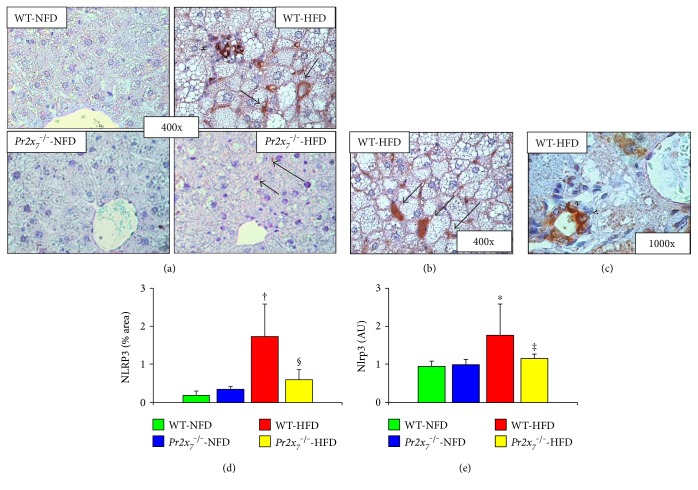
Hepatic NLRP3 expression. Immunohistochemistry for NLRP3 in liver sections from representative animals ((a) original magnification 400x, additional staining pattern features in HFD-fed WT mice in (b) original magnification 400x, and (c) original magnification 1000x oil immersion) and quantification of liver NLRP3 protein content (d) and *Nlrp3* mRNA expression (e) in NFD- and HFD-fed WT mice and *P2x_7_r*^−/−^ mice (mean ± SD; *n* = 4–6 per group). # = inflammatory cell infiltrate around degenerating hepatocytes; → = sinusoidal lining cells, especially in dilated sinusoidal spaces; > = bile duct epithelilal cells. ^∗^*P* < 0.001 or ^†^*P* < 0.01 versus the corresponding NFD-fed mice; ^‡^*P* < 0.001 or ^§^*P* < 0.05 versus WT mice. NLRP3 = nucleotide-binding and oligomerization domain (NOD), leucine-rich repeat and pyrin domain containing 3; *Nlrp3 =* NLRP3 gene; NFD = normal-fat diet; HFD = high-fat diet; WT = wild type; *P2x_7_r*^−/−^ = knockout for purinergic receptor 2X_7_ gene.

**Figure 6:**
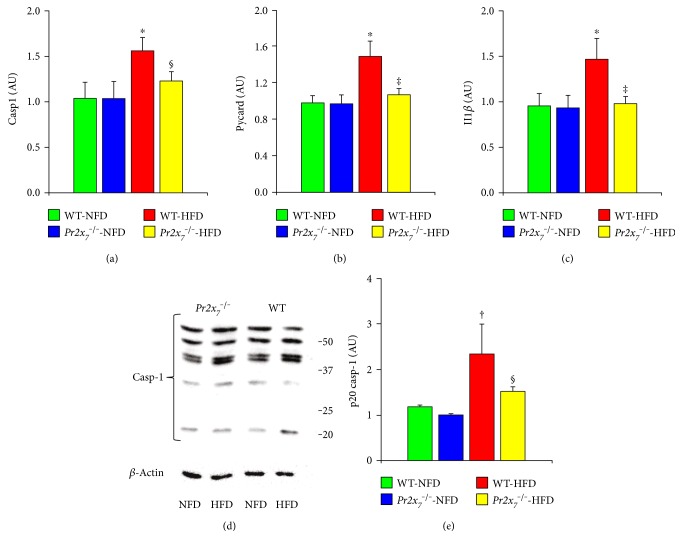
Hepatic NLRP3 inflammasome expression and activation. Liver mRNA expression of *Casp1* (a), *Pycard* (b), and *Il1β* (c); Western blot analysis of caspase-1 and housekeeping *β*-actin in liver protein extracts from representative animals (d) and quantification of 20 kDa subunit (active caspase-1) relative to *β*-actin (e) in NFD- and HFD-fed WT mice and *P2x_7_r*^−/−^ mice (mean ± SD; *n* = 4–6 per group). ^∗^*P* < 0.001 or ^†^*P* < 0.01 versus the corresponding NFD-fed mice; ^‡^*P* < 0.001 or ^§^*P* < 0.05 versus WT mice. NLRP3 = nucleotide-binding and oligomerization domain (NOD), leucine-rich repeat and pyrin domain containing 3; *Casp1 =* caspase-1 gene*; Pycard =* PYD-CARD adaptor protein apoptosis-associated speck-like protein containing a CARD gene; *Il1β* = interleukin- (IL-) 1*β* gene; NFD = normal-fat diet; HFD = high-fat diet; WT = wild type; *P2x_7_r*^−/−^ = knockout for purinergic receptor 2X_7_ gene.

Staining for PR2X_7_ was observed at both the hepatocyte and sinusoidal level in HFD-fed WT mice, whereas it was only faint in those receiving a NFD (Figures 4(a) and 4(b)). Likewise, liver mRNA expression of *Pr2x_7_* was significantly higher in HFD- versus NFD-fed WT animals, as assessed by RT-PCR (Figure 4(c)). Levels of eATP increased significantly in HFD-fed WT and to a lesser extent *Pr2x_7_*^−/−^ mice (Figure 4(d)). Also, NLRP3 protein expression was markedly increased in the liver of WT-HFD mice, as shown by the de novo appearance of a strong positive staining for NLRP3 in cells lining the hepatic sinusoids and bile ducts and in infiltrating inflammatory cells, but not in hepatocytes. Conversely, virtually no NLRP3 staining was observed in *PR2X_7_*^−/−^-HFD animals (Figures 5(a) and 5(d)). Consistently, *Nlrp3* mRNA levels were significantly increased in the liver of WT, but not *Pr2x_7_*^−/−^ mice fed with a HFD (Figure 5(e)). Similar changes were observed in the hepatic gene expression of *Pycard*, *Casp1*, and *Il1β*, which were increased in WT, but no or significantly less in *Pr2x_7_*^−/−^ on a HFD (Figures 6(a), 6(b), and 6(c)). Finally, Western blot analysis showed that the 20 kDa subunit (active caspase-1) was significantly increased in response to the HFD in WT, but not in *Pr2x_7_*^−/−^ mice (Figures 6(d) and 6(e)).

### 3.5. Cell Culture Experiments

**Figure 7:**
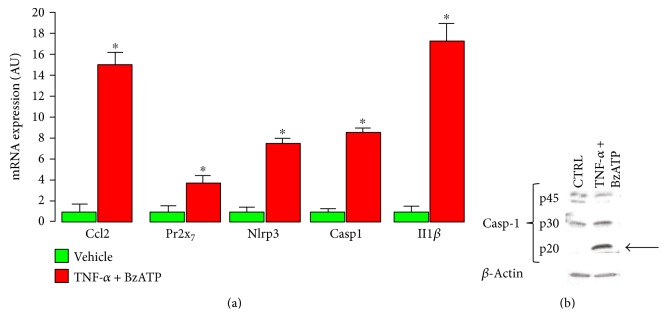
Activation of NLRP3 inflammasome in LSECs. Quantification of mRNA expression for *Ccl2* (a), *Pr2x_7_* (b), *Nlrp3* (c), *Casp1* (d), and *Il1β* (e) and Western blot analysis of caspase-1 and housekeeping *β*-actin in representative protein extracts (f) from LSECs exposed to vehicle or 100 ng/ml TNF-*α* for 24 hours, followed by 0.2 mM BzATP for 40 min (mean ± SD; *n* = 3 in triplicate). ^∗^*P* < 0.001 versus vehicle. NLRP3 = nucleotide-binding and oligomerization domain (NOD), leucine-rich repeat and pyrin domain containing 3; LSECs = liver sinusoidal endothelial cells; *Ccl2* = monocyte chemoattractant protein-1 (MCP-1) gene; *Pr2x_7_* = purinergic receptor 2X_7_ gene; *Nlrp3* = NLRP3 gene; *Casp1 =* caspase-1 gene*; Il1β* = interleukin-1*β* gene; TNF-*α* = tumor necrosis factor-*α*; BzATP = 2′(3′)-O-(4-benzoylbenzoyl)ATP.

Exposure of LSECs to TNF-*α* + Bz-ATP resulted in a marked upregulation of the gene expression levels of *Ccl2*, *Pr2x_7_*, *Nlrp3*, *Casp1*, and *Il1β* (Figure 7(a)) and in the activation of caspase-1, as shown by the appearance of a 20 kDa band at Western blot analysis (Figure 7(b)).

## 4. Discussion

This study provides the experimental evidence that *Pr2x_7_* gene deletion results in attenuation of NASH induced by a HFD, a well-established model of the human metabolic syndrome, as well as in blunted expression and activation of the NLRP3 inflammasome, which may ultimately mediate protection from liver injury.

The protective effect of *Pr2x_7_* gene deletion toward NASH is attested by the decreased inflammation, hepatocyte degeneration, and fibrosis at histological examination and the reduced or abolished increment in the protein and/or gene expression of several proinflammatory, prooxidant, and profibrotic markers. These results are consistent with a previous report showing that *Pr2x_7_* gene deletion attenuates hepatic inflammation and fibrosis in different animal models of NASH [8] and also with the findings that lack or blockade of PR2X_7_ prevents other experimentally induced liver diseases [9–11].

Interestingly, also hepatic fat accumulation was lower in *Pr2x_7_*^−/−^ than in WT mice fed with a HFD, a finding attributable to a favorable effect of *Pr2x_7_* gene knockout on the expression of transcription factors and enzymes of lipid metabolism. In particular, the upregulation of those involved in de novo lipogenesis, such as *Srebf1*, *Ppara*, *Pparg*, *Nr1h3*, *Acaca*, and *Fasn*, was significantly blunted in *Pr2x_7_*^−/−^ mice, as compared with WT animals. These data support the concept that the protection afforded by *Pr2x_7_* gene deletion was related not only to reduction of the proinflammatory and profibrotic response to the HFD but also to decreased lipotoxicity driving hepatocyte injury and release of danger signals such as eATP, which activate the innate immune system. This interpretation is consistent with the lower levels of eATP detected in the liver of *Pr2x_7_*^−/−^ versus WT mice on a HFD and also with the finding that FFAs (and glucose) are capable of upregulating *Pr2x_7_* [29], thus implying that the activation of purinergic signaling may occur at an early stage in HFD-fed mice, prior to the occurrence of inflammation-dependent hepatocyte degeneration and death.

The attenuation of NAFLD/NASH observed in *PR2x7*^−/−^ mice could not be attributed to a better metabolic profile. In fact, a reduced liver injury was observed not only in animals of the GlaxoSmithKline strain, which appeared to be partly protected from metabolic dysfunction induced by the HFD feeding, but also in those of the Pfizer strain, which did not show any improvement in metabolic parameters. The different metabolic responses observed in the two strains are in keeping with previous studies reporting that expression of splice variants which escape inactivation and maintain function, though to a reduced extent, may occur in selected tissue of *Pr2x_7_*^−/−^mice from both strains [23, 24]. In addition, the finding that mice of the Pfizer strain developed the characteristic features of the metabolic syndrome, including obesity, dyslipidemia, hyperglycemia, and insulin resistance, to the same extent as their corresponding WT animals, is consistent with a previous report by Sun et al. In fact, these authors showed no change in metabolic phenotype (and adipose tissue inflammasome activation) in mice from this strain, though they did not referred these unexpected results to splice variants of *Pr2x_7_* transcripts [30].

The association of *Pr2x_7_* gene deletion with blunted hepatic activation of the NLRP3 inflammasome points to a major role of purinergic signaling through PR2X_7_ in triggering of this process in the liver of HFD-fed mice. All the three not mutually exclusive models proposed for inflammasome activation might have been responsible for the activation of NLRP3 inflammasome in these animals [31]. In fact, dead hepatocytes may have released eATP [9] and uric acid [32], thus inducing pore formation via PR2X_7_ and frustrated phagocytosis with consequent lysosomal disintegration and release of proteases, respectively [31]. In addition, multiple signals may have caused generation of reactive oxygen species (ROS) via NADPH oxidase or mitochondrial sources, which is thought to represent a common final pathway leading to inflammasome activation [31]. These signals include glucose [33], saturated FFAs such as palmitate [34], the lipotoxic FFA metabolite ceramide [35], and modified lipoproteins such as oxidized LDLs, which may also act via formation of cholesterol crystals [36]. In addition, Chatterjee et al. recently showed that, in Kupffer cells, PR2X_7_ mediates NADPH oxidase activation by upregulating the expression of the p47 phox subunit and p47 phox binding to the membrane subunit, gp91 phox [37], consistent with our finding of a reduced liver expression of NCF1 (p47 phox) in *Pr2x_7_*^−/−^ animals. Other possible mechanisms implicated in the PR2X_7_-mediated activation of the NLRP3 inflammasone include the induction of endoplasmic reticulum stress [38] and the impairment of autophagy [39], which have been implicated in the development of NASH [8, 40]. Finally, the release of microvesicles from fat-laden hepatocytes may promote activation of NLRP3 in surrounding hepatocytes via a paracrine mechanism [41].

**Figure 8:**
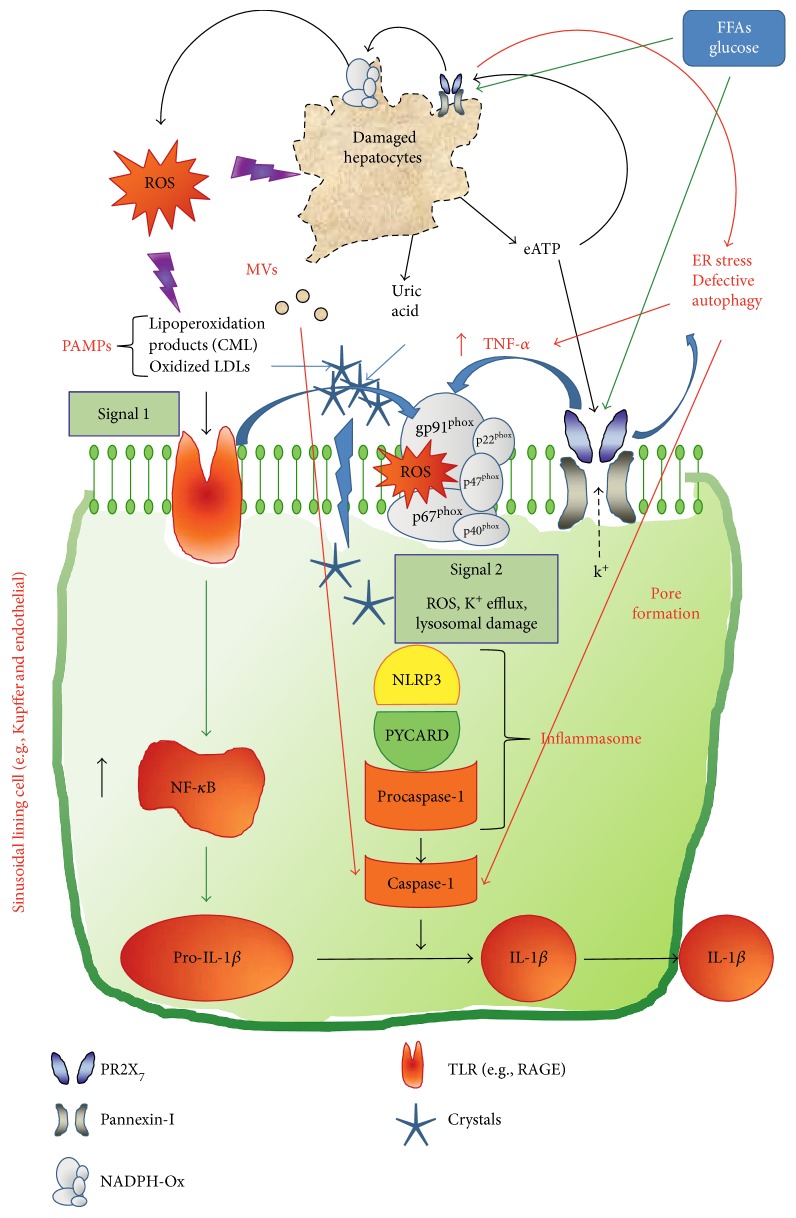
Potential mechanisms involved in PR2X_7_-mediated expression/activation of the NLRP3 inflammasome. CML = N*^ε^*-(carboxymethyl)lysine; DAMPs = damage-associated molecular patterns; eATP = extracellular ATP; ER = endoplasmic reticulum; FFAs = free fatty acids; IL-1*β* = interleukin-1*β*; LDLs = low density lipoprotein; MVs = microvesicles; NADPH ox = NADPH oxidase; NF-*κ*B = nuclear factor-*κ*B; NLRP3 = nucleotide-binding and oligomerization domain (NOD), leucine-rich repeat and pyrin domain containing 3; PR2X_7_ = purinergic receptor 2X_7_; PYCARD = the PYD-CARD adaptor protein apoptosis-associated speck-like protein containing a caspase recruitment domain; RAGE = receptor for advanced glycation end products; ROS = reactive oxygen species; TLR = toll-like receptor; TNF-*α* = tumor necrosis factor-*α*.

As previously shown at the kidney level, also the expression of NLRP3 inflammasome components was blunted in liver tissue of HFD-fed *Pr2x_7_*^−/−^ mice, thus suggesting that stimulation of PR2X_7_ is involved also in priming the inflammasome through activation of nuclear factor-*κ*B (NF-*κ*B) via multiple mechanisms. Activation of NADPH oxidase by PR2X_7_-mediated increase of TNF-*α* levels through autophagy-linked inflammation may play a major role in this respect [8, 37]. Moreover, the decreased levels of CML and *Ager* and the improved lipid metabolism detected in the liver of these mice suggest that PR2X_7_ also modulates the activation of NF-*κ*B induced by carbonyl stress [42] and FFAs [43] via oxidant-dependent mechanisms. Activation of procaspase-1 and release of mature IL-1*β* induced by PR2X_7_-mediated inflammasome activation might also be important in sustaining NF-*κ*B activation via induction of TNF-*α* [44].  Figure 8 summarizes the potential mechanisms involved in PR2X7-mediated expression/activation of the NLRP3 inflammasome.

The finding that both the expression and the activation of the NLRP3 inflammasome were blunted in *Pr2x_7_*^−/−^ mice supports the hypothesis that this effect of *Pr2x_7_* gene deletion was a major mechanism mediating protection from NASH. This interpretation is consistent with previous observations showing that *Nlrp3* gene deletion [18] or NLRP3 blockade [17] attenuates hepatic inflammation and fibrosis in animal models of NASH, and also with the evidence that these strategies are effective in attenuating other experimentally induced liver diseases [14].

A debated issue is the contribution of resident cells to inflammasome activation and inflammasome-mediated tissue injury in inflammatory and fibrotic disorders. Indeed, in the liver of WT-HFD mice, the NLRP3 inflammasome was expressed in both resident and nonresident cells. In addition, inflammasome activation was induced in LSECs challenged with TNF-*α* + BzATP, that is, a primer and an activator of this multiprotein platform. These data are consistent with the concept that also resident cells are involved, as previously shown in the kidneys of these animals [16]. Csak et al. found that both bone marrow- (BM-) derived and non-BM-derived cells contributed to NLRP3 inflammasome activation in a myeloid differentiation primary response gene 88- (MyD88-) dependent manner in NASH induced by MCD diet, though only BM-derived cell-specific MyD88-deficiency attenuated liver injury [45]. Conversely, Wree et al. reported that acceleration of NASH was lower in BM-specific than in global *Nl3p3* knockin mice, thus pointing to an important contribution of resident cells to liver injury [20].

A limitation of this study is the use of LSECs only to assess whether PR2X_7_-dependent activation of the NLRP3 inflammasome occurs also at the level of resident liver cells. However, our aim was to test this hypothesis in a nonimmune cell type of the liver other than hepatocytes, which did not show any NLRP3 protein expression at IHC.

## 5. Conclusion

These data demonstrate a major contribution of PR2X_7_, possibly via activation of the NLRP3 inflammasome, in hepatic inflammation and injury driving transition from steatosis to NASH in the context of NAFLD. This study identifies P2X_7_R and NLRP3 as novel therapeutic targets for liver disease associated with metabolic disorders.

## Supplementary Material

Supplemental Table 1. Primary and secondary antibodies used in the immunohistochemistry and western blot studies. Supplemental Table 2. TaqMan Assays used in quantitative real time PCR.

## Figures and Tables

**Table 1:** Metabolic parameters and liver enzymes. Body weights and levels of glucose, insulin, HOMA-IR index, cholesterol, triglycerides, FFAs, AST, and ALT in WT and *Pr2x_7_^−/−^* mice fed with a NFD or a HFD (mean ± SD; *n* = 7 per group).

	WT-NFD	*Pr2x_7_* ^−/−^-NFD	WT-HFD	*Pr2x_7_* ^−/−^-HFD
Body weight, g	31.8 ± 2.3	32.6 ± 1.9	41.2 ± 5.5^∗^	40.6 ± 1.5^∗^
Glucose, mmol·l^−1^	5.56 ± 0.88	5.63 ± 0.73	9.01 ± 0.71^∗^	8.85 ± 1.08^∗^
Insulin, pmol·l^−1^	63.9 ± 18.7	58.3 ± 11.5	158.1 ± 18.1^∗^	151.9 ± 19.1^∗^
HOMA-IR	2.21 ± 0.79	2.05 ± 0.56	8.84 ± 1.32^∗^	8.38 ± 1.86^∗^
Cholesterol, mmol·l^−1^	2.64 ± 0.31	2.62 ± 0.54	3.93 ± 0.62^∗^	3.82 ± 0.31^∗^
Triglycerides, mmol·l^−1^	0.56 ± 0.21	0.57 ± 0.17	1.78 ± 0.52^∗^	1.42 ± 0.29^∗^
FFA, mEq·l^−1^	0.33 ± 0.05	0.31 ± 0.06	0.95 ± 0.11^∗^	0.83 ± 0.09^∗^
AST, UI·l^−1^	33.6 ± 5.6	31.3 ± 4.6	87.1 ± 12.3^∗^	55.9 ± 7.1^∗^^†^
ALT, UI·l^−1^	22.6 ± 7.2	18.6 ± 3.6	111.4 ± 18.4^∗^	58.3 ± 15.7^∗^^†^

HOMA-IR = homeostasis model assessment-insulin resistance; FFAs = free fatty acids; AST = aspartate transaminase; ALT = alanine transaminase; WT = wild type; *Pr2x_7_^−/−^* = knockout for purinergic receptor X2_7_ gene; NFD = normal-fat diet; HFD = high-fat diet. ^∗^*P* < 0.001 versus the corresponding NFD-fed mice; ^†^*P* < 0.001 versus WT mice.

**Table 2:** Liver inflammation, fibrosis, and lipid metabolism. Liver expression of genes of inflammation, fibrosis, and lipid metabolism in WT and *Pr2x_7_^−/−^* mice fed with a NFD or a HFD (mean ± SD; *n* = 4–7 per group). ^∗^*P* < 0.01 versus the corresponding NFD mice; ^†^*P* < 0.05 versus the corresponding WT mice.

	WT-NFD	*Pr2x_7_^−/−^*-NFD	WT-HFD	*Pr2x_7_^−/−^*-HFD
*Inflammation*				
*Ccl2*	0.90 ± 0.30	0.98 ± 0.28	2.79 ± 0.79^∗^	1.56 ± 0.49^‡^
*Tnfa*	1.07 ± 0.22	1.03 ± 0.58	2.18 ± 0.71^†^	1.13 ± 0.31^§^
*Ifng*	1.02 ± 0.13	1.03 ± 0.06	1.27 ± 0.20	1.18 ± 0.11
*Cxcr3*	0.97 ± 0.13	1.06 ± 0.26	2.86 ± 1.07^∗^	1.65 ± 0.50^#^
*Adgre1*	1.08 ± 0.26	1.11 ± 0.55	2.54 ± 0.60^∗^	1.62 ± 0.37^§^
*Ddit3*	1.01 ± 0.07	0.98 ± 0.08	1.93 ± 0.20^∗^	1.17 ± 0.30^‡^
*Ager*	1.02 ± 0.07	1.03 ± 0.07	1.93 ± 0.18^∗^	1.28 ± 0.38^§^
*Fibrosis*				
*Fn1*	0.98 ± 0.06	0.92 ± 0.07	1.68 ± 0.29^∗^	1.09 ± 0.03^‡^
*Cola1a1*	1.00 ± 0.17	0.93 ± 0.06	2.33 ± 0.49^∗^	1.34 ± 0.27^§^
*Tgfβ*	1.08 ± 0.13	1.01 ± 0.22	1.84 ± 0.26^∗^	1.50 ± 0.26^†#^
*Lipid metabolism*				
*Srebf1*	0.91 ± 0.15	0.99 ± 0.22	3.80 ± 0.52^∗^	1.90 ± 0.27^∗^^‡^
*Ppara*	0.97 ± 0.21	0.93 ± 0.07	1.90 ± 0.52^†^	1.45 ± 0.33
*Pparg*	0.92 ± 0.16	0.93 ± 0.15	2.52 ± 0.71^∗^	1.47 ± 0.13^§^
*Nr1h3*	0.96 ± 0.09	0.92 ± 0.12	1.47 ± 0.14^∗^	1.24 ± 0.10^†#^
*Nr1h2*	1.09 ± 0.12	1.12 ± 0.14	1.22 ± 0.21	1.23 ± 0.19
*Acaca*	0.87 ± 0.15	0.92 ± 0.11	1.21 ± 0.06^∗^	0.97 ± 0.08^‡^
*Fasn*	0.96 ± 0.15	0.99 ± 0.20	1.53 ± 0.11^∗^	1.19 ± 0.17^§^
*Cpt1*	0.95 ± 0.09	1.02 ± 0.19	2.45 ± 0.39^∗^	1.64 ± 0.28^∗^^‡^
*Acox1*	0.95 ± 0.08	0.90 ± 0.14	1.07 ± 0.26	1.00 ± 0.22
*Hmgcr*	0.99 ± 0.05	0.98 ± 0.07	1.10 ± 0.16	1.05 ± 0.16
*Mttp*	0.91 ± 0.10	0.98 ± 0.08	0.91 ± ±0.18	0.99 ± 0.23

WT = wild type; *Pr2x_7_^−/−^* = knockout for purinergic receptor X2_7_ gene; NFD = normal-fat diet; HFD = high-fat diet; *Ccl2* = monocyte chemoattractant protein-1 (MCP-1) gene; *Tnfa* = tumor necrosis factor-*α* gene; *Ifng* = interferon-*γ* gene; *Cxcr3* = CX chemokine receptor 3 gene; *Adgre1* = F4/80 gene; *Ddit3* = m CCAAT/enhancer-binding protein (*C/EBP*) homologous protein (CHOP) gene; *Ager* = receptor for AGEs (RAGE) gene; *Fn1* fibronectin gene; *Col1a1* = collagen I gene; *tgfb1* = transforming growth factor- (TGF-) *β*1 gene; *Srebf1* = sterol regulatory element-binding transcription factor 1c gene; *Ppara* = peroxisome proliferator-activated receptor (PPAR) *α* gene; *Pparg* = peroxisome proliferator-activated receptor (PPAR) *γ* gene; *Nr1h3* = liver X receptor- (LRX-) *α* gene; *Nr1h2* = LXR-*β* gene; *Acaca* = acetyl-CoA carboxylase gene; *Fasn* = fatty acid synthase gene; *Cpt1a* = carnitine palmitoyltransferase- (CPT-) I gene; *Acox1* = acyl-CoA oxidase 1 gene; *Hmgcr* = hydroxymethylglutaryl-CoA reductase gene; *Mttp* = microsomal triglyceride transfer protein gene. ^∗^*P* < 0.001 or ^†^*P* < 0.01 versus the corresponding NFD-fed mice; ^‡^*P* < 0.001, ^§^*P* < 0.01, or ^#^*P* < 0.05, versus WT mice.

## References

[1] Younossi Z. M., Koenig A. B., Abdelatif D., Fazel Y., Henry L., Wymer M. (2016). Global epidemiology of nonalcoholic fatty liver disease-meta-analytic assessment of prevalence, incidence, and outcomes. *Hepatology*.

[2] Angulo P. (2002). Nonalcoholic fatty liver disease. *The New England Journal of Medicine*.

[3] Hardy T., Oakley F., Anstee Q. M., Day C. P. (2016). Nonalcoholic fatty liver disease: pathogenesis and disease spectrum. *Annual Review of Pathology: Mechanisms of Disease*.

[4] Neuschwander-Tetri B. A. (2010). Hepatic lipotoxicity and the pathogenesis of nonalcoholic steatohepatitis: the central role of nontriglyceride fatty acid metabolites. *Hepatology*.

[5] Fuchs M., Sanyal A. J. (2012). Lipotoxicity in NASH. *Journal of Hepatology*.

[6] Burnstock G., Vaughn B., Robson S. C. (2014). Purinergic signalling in the liver in health and disease. *Purinergic Signalling*.

[7] Chatterjee S., Das S. (2015). P2X7 receptor as a key player in oxidative stress-driven cell fate in nonalcoholic steatohepatitis. *Oxidative Medicine and Cellular Longevity*.

[8] Das S., Seth R. K., Kumar A. (2013). Purinergic receptor X_7_ is a key modulator of metabolic oxidative stress-mediated autophagy and inflammation in experimental nonalcoholic steatohepatitis. *American Journal of Physiology Gastrointestinal and Liver Physiology*.

[9] Hoque R., Sohail M. A., Salhanick S. (2012). P_2_X_7_ receptor-mediated purinergic signaling promotes liver injury in acetaminophen hepatotoxicity in mice. *American Journal of Physiology Gastrointestinal Liver Physiology*.

[10] Huang C., Yu W., Cui H. (2014). P2X7 blockade attenuates mouse liver fibrosis. *Molecular Medicine Reports*.

[11] Tung H.-C., Lee F.-Y., Wang S.-S. (2015). The beneficial effects of P2X_7_ antagonism in rats with bile duct ligation-induced cirrhosis. *PLoS One*.

[12] Pelegrin P., Surprenant A. (2006). Pannexin-1 mediates large pore formation and interleukin-1β release by the ATP-gated P2X7 receptor. *The EMBO Journal*.

[13] Schroder K., Tschopp J. (2010). The inflammasomes. *Cell*.

[14] Szabo G., Csak T. (2012). Inflammasomes in liver diseases. *Journal of Hepatology*.

[15] Lamkanfi M. (2011). Emerging inflammasome effector mechanisms. *Nature Reviews Immunology*.

[16] Solini A., Menini S., Rossi C. (2013). The purinergic 2X_7_ receptor participates in renal inflammation and injury induced by high-fat diet: possible role of NLRP3 inflammasome activation. *The Journal of Pathology*.

[17] Mridha A. R., Wree A., Robertson A. A. B. (2017). NLRP3 inflammasome blockade reduces liver inflammation and fibrosis in experimental NASH in mice. *Journal of Hepatology*.

[18] Wree A., McGeough M. D., Peña C. A. (2014). NLRP3 inflammasome activation is required for fibrosis development in NAFLD. *Journal of Molecular Medicine*.

[19] Henao-Mejia J., Elinav E., Jin C. (2012). Inflammasome-mediated dysbiosis regulates progression of NAFLD and obesity. *Nature*.

[20] Wree A., Eguchi A., McGeough M. D. (2014). NLRP3 inflammasome activation results in hepatocyte pyroptosis, liver inflammation, and fibrosis in mice. *Hepatology*.

[21] Solle M., Labasi J., Perregaux D. G. (2001). Altered cytokine production in mice lacking P2X_7_ receptors. *Journal of Biological Chemistry*.

[22] Chessell I. P., Hatcher J. P., Bountra C. (2005). Disruption of the P2X7 purinoceptor gene abolishes chronic inflammatory and neuropathic pain. *Pain*.

[23] Masin M., Young C., Lim K. (2012). Expression, assembly and function of novel C-terminal truncated variants of the mouse P2X7 receptor: re-evaluation of P2X7 knockouts. *British Journal of Pharmacology*.

[24] Nicke A., Kuan Y. H., Masin M. (2009). A functional P2X7 splice variant with an alternative transmembrane domain 1 escapes gene inactivation in P2X7 knock-out mice. *Journal of Biological Chemistry*.

[25] Iacobini C., Menini S., Ricci C. (2011). Galectin-3 ablation protects mice from diet-induced NASH: a major scavenging role for galectin-3 in liver. *Journal of Hepatology*.

[26] Wree A., McGeough M. D., Inzaugarat M. E. (2017). NLRP3 inflammasome driven liver injury and fibrosis: roles of IL-17 and TNF. *Hepatology*.

[27] Neuschwander-Tetri B. A., Caldwell S. H. (2003). Nonalcoholic steatohepatitis: summary of an AASLD single topic conference. *Hepatology*.

[28] Menini S., Iacobini C., Ricci C., Blasetti Fantauzzi C., Pugliese G. (2015). Protection from diabetes-induced atherosclerosis and renal disease by D-carnosine-octylester: effects of early versus late inhibition of advanced glycation end-products in *Apoe*-null mice. *Diabetologia*.

[29] Glas R., Sauter N. S., Schulthess F. T., Shu L., Oberholzer J., Maedler K. (2009). Purinergic P2X_7_ receptors regulate secretion of interleukin-1 receptor antagonist and beta cell function and survival. *Diabetologia*.

[30] Sun S., Xia S., Ji Y., Kersten S., Qi L. (2012). The ATP-P2X_7_ signaling axis is dispensable for obesity-associated inflammasome activation in adipose tissue. *Diabetes*.

[31] Strowig T., Henao-Mejia J., Elinav E., Flavell R. (2012). Inflammasomes in health and disease. *Nature*.

[32] Watanabe A., Sohail M. A., Gomes D. A. (2009). Inflammasome-mediated regulation of hepatic stellate cells. *American Journal of Physiology Gastrointestinal and Liver Physiology*.

[33] Giacco F., Brownlee M. (2010). Oxidative stress and diabetic complications. *Circulation Research*.

[34] Csak T., Ganz M., Pespisa J., Kodys K., Dolganiuc A., Szabo G. (2011). Fatty acid and endotoxin activate inflammasomes in mouse hepatocytes that release danger signals to stimulate immune cells. *Hepatology*.

[35] Kolliputi N., Galam L., Parthasarathy P. T., Tipparaju S. M., Lockey R. F. (2012). NALP-3 inflammasome silencing attenuates ceramide-induced transepithelial permeability. *Journal Cellular Physiology*.

[36] Duewell P., Kono H., Rayner K. J. (2010). NLRP3 inflammasomes are required for atherogenesis and activated by cholesterol crystals. *Nature*.

[37] Chatterjee S., Rana R., Corbett J., Kadiiska M. B., Goldstein J., Mason R. P. (2012). P2X7 receptor-NADPH oxidase axis mediates protein radical formation and Kupffer cell activation in carbon tetrachloride-mediated steatohepatitis in obese mice. *Free Radical & Biology Medicine*.

[38] Chao C. C., Chan P., Kuo C. S. (2014). Protection of differentiated neuronal NG108-15 cells from P2X7 receptor-mediated toxicity by taurine. *Pharmacological Reports*.

[39] Takenouchi T., Nakai M., Iwamaru Y. (2009). The activation of P2X7 receptor impairs lysosomal functions and stimulates the release of autophagolysosomes in microglial cells. *The Journal of Immunology*.

[40] Lebeaupin C., Proics E., de Bieville C. H. (2015). ER stress induces NLRP3 inflammasome activation and hepatocyte death. *Cell Death & Disease*.

[41] Cannito S., Morello E., Bocca C. (2017). Microvesicles released from fat-laden cells promote activation of hepatocellular NLRP3 inflammasome: a pro-inflammatory link between lipotoxicity and non-alcoholic steatohepatitis. *PLoS One*.

[42] Bierhaus A., Chevion S., Chevion M. (1997). Advanced glycation end product-induced activation of NF-ĸB is suppressed by α-lipoic acid in cultured endothelial cells. *Diabetes*.

[43] Nguyen M. T., Favelyukis S., Nguyen A. K. (2007). A subpopulation of macrophages infiltrates hypertrophic adipose tissue and is activated by free fatty acids via toll-like receptors 2 and 4 and JNK-dependent pathways. *Journal of Biological Chemistry*.

[44] Wen H., Gris D., Lei Y. (2011). Fatty acid-induced NLRP3-ASC inflammasome activation interferes with insulin signaling. *Nature Immunology*.

[45] Csak T., Pillai A., Ganz M. (2014). Both bone marrow-derived and non-bone marrow-derived cells contribute to AIM2 and NLRP3 inflammasome activation in a MyD88-dependent manner in dietary steatohepatitis. *Liver International*.

