# Supplementary material for: Deficiency of the Purinergic Receptor 2X7 Attenuates Nonalcoholic Steatohepatitis Induced by High-Fat Diet: Possible Role of the NLRP3 Inflammasome
Source: Oxid Med Cell Longev. 2017 Nov 15;2017:8962458. doi: 10.1155/2017/8962458 (PMC5705892; doi:10.1155/2017/8962458)
Supplement: Supplementary file 1 — Supplemental Table 1. Primary and secondary antibodies used in the immunohistochemistry and western blot studies. Supplemental Table 2. TaqMan Assays used in quantitative real time PCR. [file 8962458.f1.doc]

**Supplemental Table 1.** Primary and secondary antibodies used in the immunohistochemistry and western blot studies.

| **Target** | **Primary antibody** | **Secondary antibody** |
| --- | --- | --- |
| **CML** | mouse monoclonal to CML (biotinylated) (Wako, Neuss, Germany) | biotinylated goat polyclonal to mouse IgG H&L (ab6788, Abcam, Cambridge, UK) |
| **NCF1** | rabbit polyclonal to NCF1 (ab137950, Abcam) | biotinylated goat anti-rabbit immunoglobulins |
| **PR2X_7_** | rabbit polyclonal to P2X_7_R (ab48871, Abcam) | polyclonal goat anti-rabbit immunoglobulins/HRP (P0448, Dako, Carpinteria, CA, USA) |
| **NLRP3** | rabbit polyclonal to NLRP3 (HPA012878, Sigma , St.Louis, MO, USA) | polyclonal goat anti-rabbit immunoglobulins/HRP (P0448, Dako) |
| **Caspase-1** | rabbit polyclonal to caspase-1 (06-503, Millipore) | polyclonal goat anti-rabbit immunoglobulins/HRP (P0448, Dako) |
| **Β-actin** | mouse monoclonal to β-actin (A5441, Sigma) | polyclonal goat anti-mouse immunoglobulins/HRP (P0447, Dako) |

CML = N^ε^-(carboxymethyl)lysine; NCF1 = neutrophil cytosol factor 1; PR2X_7_ = purinergic receptor 2X_7_; NLRP3 = nucleotide-binding and oligomerization domain (NOD), leucine-rich repeat and pyrin domain containing 3.

**Supplemental Table 2.** TaqMan Assays used in quantitative real time PCR.

| **Mouse TaqMan Assay** | **Code** |
| --- | --- |
| *Ccl2* | Mm00441242_m1 |
| *Tnfa* | Mm99999058_m1 |
| *Ifng* | Mm01168134_m1 |
| *Cxcr3* | Mm00438354_m1 |
| *Adgre1* | Mm00802529_m1 |
| *Ddit3* | Mm01135937_g1 |
| *Ager* | Mm00545815_m1 |
| *FN1* | Mm00692666_m1 |
| *Col1a1* | Mm00801666_g1 |
| *Tgfβ1* | Mm01178820_m1 |
| *Srebf1* | Mm00550338_m1 |
| *Ppara* | Mm00440939_m1 |
| *Pparg* | Mm00440940_m1 |
| *Nr1h3* | Mm00443451_m1 |
| *Nr1h2* | Mm00437262_m1 |
| *Acaca* | Mm01304257_m1 |
| *Fasn* | Mm00662319_m1 |
| *Cpt1a* | Mm01231183_m1 |
| *Acox1* | Mm01246834_m1 |
| *Hmgcr* | Mm01282499_m1 |
| *Mttp* | Mm00435015_m1 |
| *Pr2x_7_* | Mm00440578_m1 |
| *Nlrp3* | Mm00840904_m1 |
| *Pycard* | Mm00445747_g1 |
| *Casp1* | Mm00438023_m1 |
| *Il1β* | Mm00434228_m1 |
| *Actb* | Mm00607939_s1 |

*Ccl2* = monocyte chemoattractant protein-1 (MCP-1) gene; *Tnfa* = tumor necrosis factor-α gene; *Ifng* = interferon-γ gene; *Cxcr3* = CX chemokine receptor 3 gene; *Adgre1* = F4/80 gene; *Ddit3* = m CCAAT/enhancer binding protein (*C/EBP*) homologous protein (CHOP) gene; *Ager* = receptor for AGEs (RAGE) gene; *Fn1* fibronectin gene; *Col1a1* = collagen I gene; *tgfb1* = transforming growth factor (TGF)-β1 gene; *Srebf1* = sterol regulatory element binding transcription factor 1c gene; *Ppara* = peroxisome proliferator-activated receptor (PPAR) α gene; *Pparg* = PPARγ gene; *Nr1h3* = liver X receptor (LRX)-α gene; *Nr1h2* = LXR-β gene; *Acaca* = acetyl-CoA carboxylase gene; *Fasn* = fatty acid synthase gene; *Cpt1a* = carnitine palmitoyltransferase (CPT)-I gene; *Acox1* = acyl-CoA oxidase 1 gene; *Hmgcr* = hydroxymethylglutaryl-CoA reductase gene; *Mttp* = microsomal triglyceride transfer protein gene; *Pr2x_7_* = purinergic receptor 2X_7_ gene; *Nlrp3*  = nucleotide-binding and oligomerization domain (NOD), leucine-rich repeat and pyrin domain containing 3 gene; *Casp1 =* caspase-1 gene*; Pycard =* PYD-CARD adaptor protein apoptosis-associated speck-like protein containing a CARD gene; *Il1β* = interleukin (IL)-1β gene; *Actb* = β-actin gene.
